# Voltammetric sensing of recombinant viral dengue virus 2 NS1 based on Au nanoparticle–decorated multiwalled carbon nanotube composites

**DOI:** 10.1007/s00604-020-04339-y

**Published:** 2020-06-02

**Authors:** Quentin Palomar, XingXing Xu, Chantal Gondran, Michael Holzinger, Serge Cosnier, Zhen Zhang

**Affiliations:** 1 Division of Solid-State Electronics, Department of Engineering Sciences, The Ångström Laboratory, Uppsala University, P.O. Box 534, SE-751 21 Uppsala, Sweden; 2 CNRS, DCM UMR 5250, Université Grenoble Alpes, F 38000 Grenoble, France

**Keywords:** Electrochemical biosensor, Differential pulste voltammetry, Carbon nanotube, Gold nanoparticles, Dengue toxin

## Abstract

A homemade gold electrode is modified with a carbon nanotubes/gold nanoparticles nanocomposite to perform selective and sensitive electrochemical detection of dengue toxin. This nanostructured composite offers a large specific surface and a reactive interface allowing the immobilization of biological material. Dengue antibodies are immobilized on gold nanoparticles via covalent bonding for dengue toxin detection. The porous tridimensional network of carbon nanotubes and gold nanoparticles enhances the electrochemical signal and the overall performance of the sensor. After optimization, the system exhibits a high sensitivity of − 0.44 ± 0.01 μA per decade with wide linear range between 1 × 10^−12^ and 1 × 10^−6^ g/mL at a working potential of 0.22 V vs Ag/AgCl. The extremely low detection limit (3 × 10^−13^ g/mL) ranks this immunosensor as one of the most efficient reported in the literature for the detection of recombinant viral dengue virus 2 NS1. This biosensor also offers good selectivity, characterized by a low response to various non-specific targets and assays in human serum. The outstanding performances and the reproducibility of the system place the biosensor developed among the best candidates for future medical applications and for early diagnosis of dengue fever.

**Electronic supplementary material:**

The online version of this article (10.1007/s00604-020-04339-y) contains supplementary material, which is available to authorized users.

**Figure:**
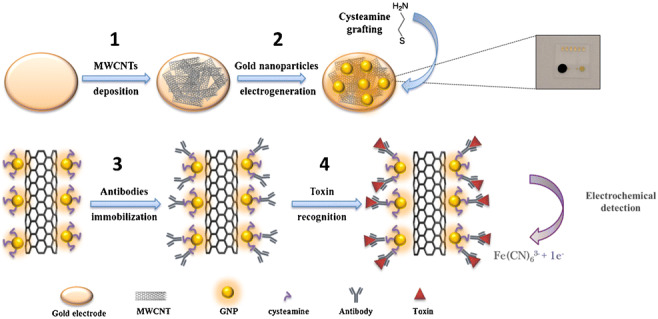
Graphical abstract

## Introduction

It is well documented that the field of electrochemical biosensors has been growing in popularity in recent years [1–3]. In this context, nanostructured electrodes have emerged as a promising candidate to be used as the sensing interface in such systems. Nanostructured electrodes, thanks to intrinsic properties of the nanomaterials and the large specific surface are that they offer [4], can lead to increased sensitivity, faster electron transfer and higher current densities. Among the different nanomaterials available to form such structures, multi-walled carbon nanotubes (MWCNTs) are popular candidates [5–7], as are gold nanoparticles (GNPs) [8, 9]. Combining the inherent properties of MWCNTs and Au-NPs will provide a sensing electrode that offers high conductivity, improved electron transfer, large effective sensing surface, good biocompatibility, and chemical properties for easy biomolecule immobilization. Different groups have investigated this approach by studying the basic electrochemical response of this system [10–12]. Wu et al. [13] developed a choline sensor using this nanomaterial and Saeedfar et al. [14] performed DNA detection. However, they used sol-gel or drop-casting processes for the modification of the electrode by nanotubes. These techniques may suffer from a lack of control over the properties of the nanostructure formed and new methods have since been developed to allow a better control over the MWCNTs layer production [15]. Therefore the combination of these materials for the detection of complex targets of interest with a well-controlled nanostructure remains to be studied. The novelty of this work is thereby partly based on the development of a protocol for manufacturing a MWCNT/GNP nanocomposite with controlled and tunable properties such as the film thickness, the size of the GNP and the conductivity.

Another important point to take into account during the construction of an electrochemical biosensor is the analytical technique used. In the case of immunosensors, where the recognition event does not lead to an enzymatically generated redox active product, it is common to monitor the biological recognition event through the signal generated by a redox probe. This can be done by monitoring the intensity of the peak current of the probe, in particular by cyclic voltammetry (CV) and differential pulse voltammetry (DPV), or by studying the impedance of the system by electrochemical impedance spectroscopy (EIS) [16].

An additional advantage of electrochemical detection is the speed and simplicity to carry out the detection, whereas in many current detection method are time-consuming and laborious (Polymerase chain reaction tests) [17]. With an electrochemical system, detection is user friendly and requires only a very small quantity of samples (~ 50 μL) and can be adapted for use in the field.

**Fig. 1:**
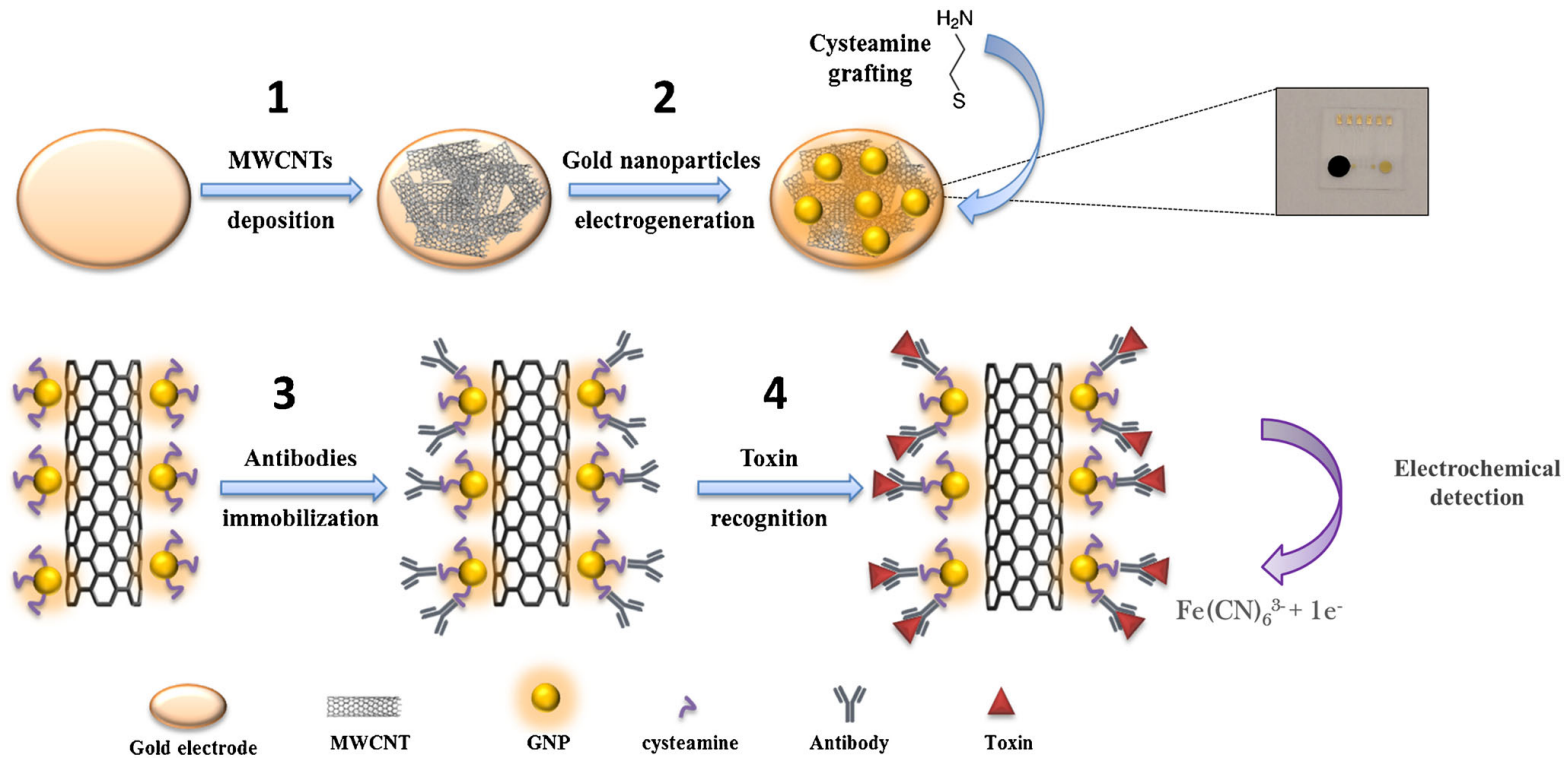
Schematic representation for the formation of the immunosensor and its operation: [1] MWCNTs modification, [2] electrogeneration of GNPs, [3] dengue antibodies immobilization via EDC/ NHS coupling, [4] toxin recognition

In this work, homemade gold electrode (GE), MWCNTs and GNPs were combined to form a nanostructured sensing platform. This electrode was functionalized by immobilizing dengue antibodies on the GNPs via covalent bonding for dengue toxin detection, and ferri/ferrocyanide (Fe (II/III)) was employed as the signal reporter. Dengue toxin has been chosen as target following the development of the disease throughout the world in recent years [18, 19]. In addition, a great demand for early diagnosis of this pathology has emerged to improve the management of patients during the first days of infection. The different steps involved during the construction of the biosensor and its operating principle are illustrated in Fig. 1.

**Fig. 2:**
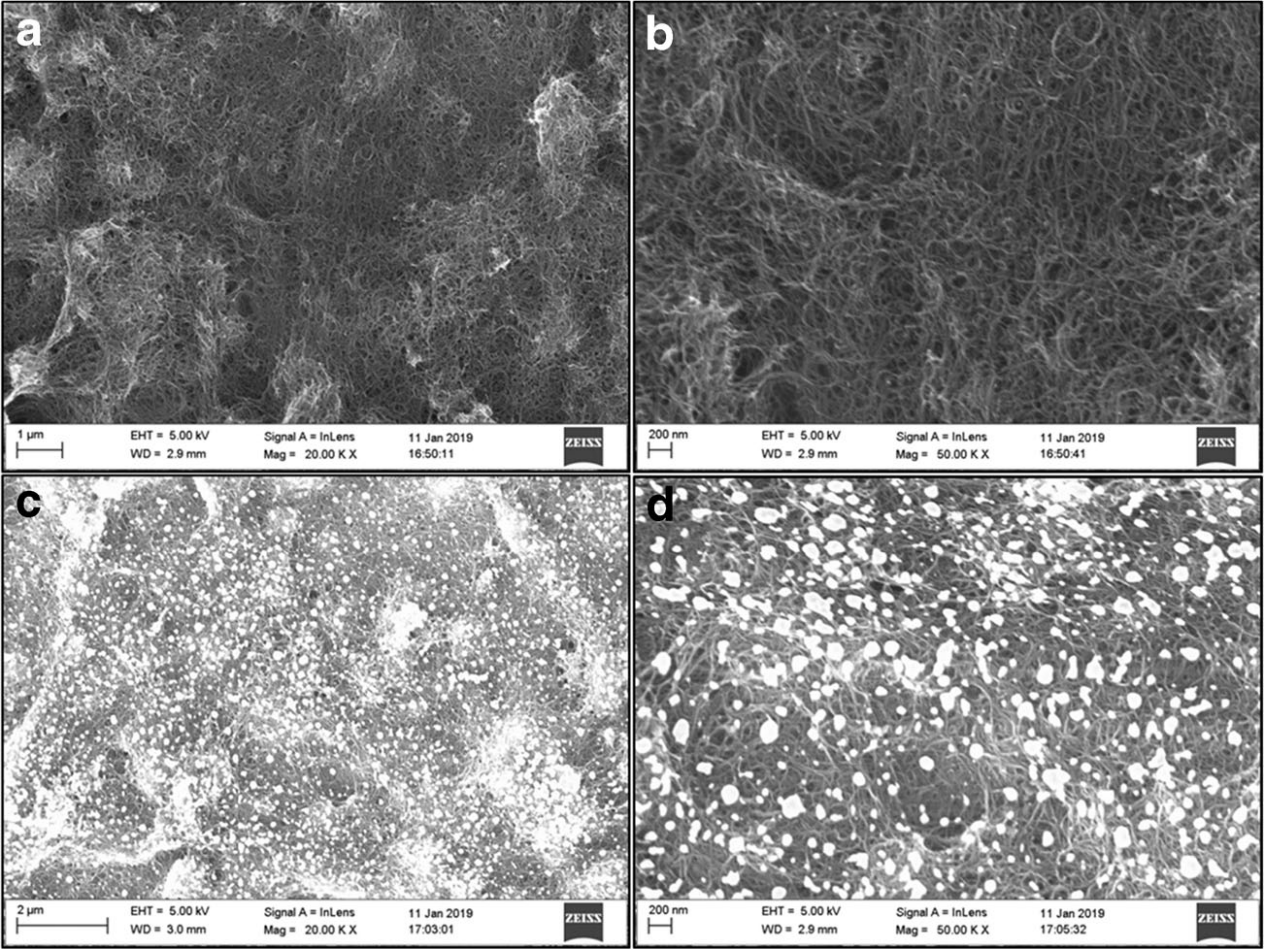
SEM images of **a** gold-MWCNTs electrode at a magnification of × 20,000, **b** gold-MWCNTs electrode at a magnification of × 50,000, **c** gold-MWCNTs-GNPs electrode at a magnification of × 20,000, and **d** gold-MWCNTs-GNPs electrode at a magnification of × 50,000

**Fig. 3:**
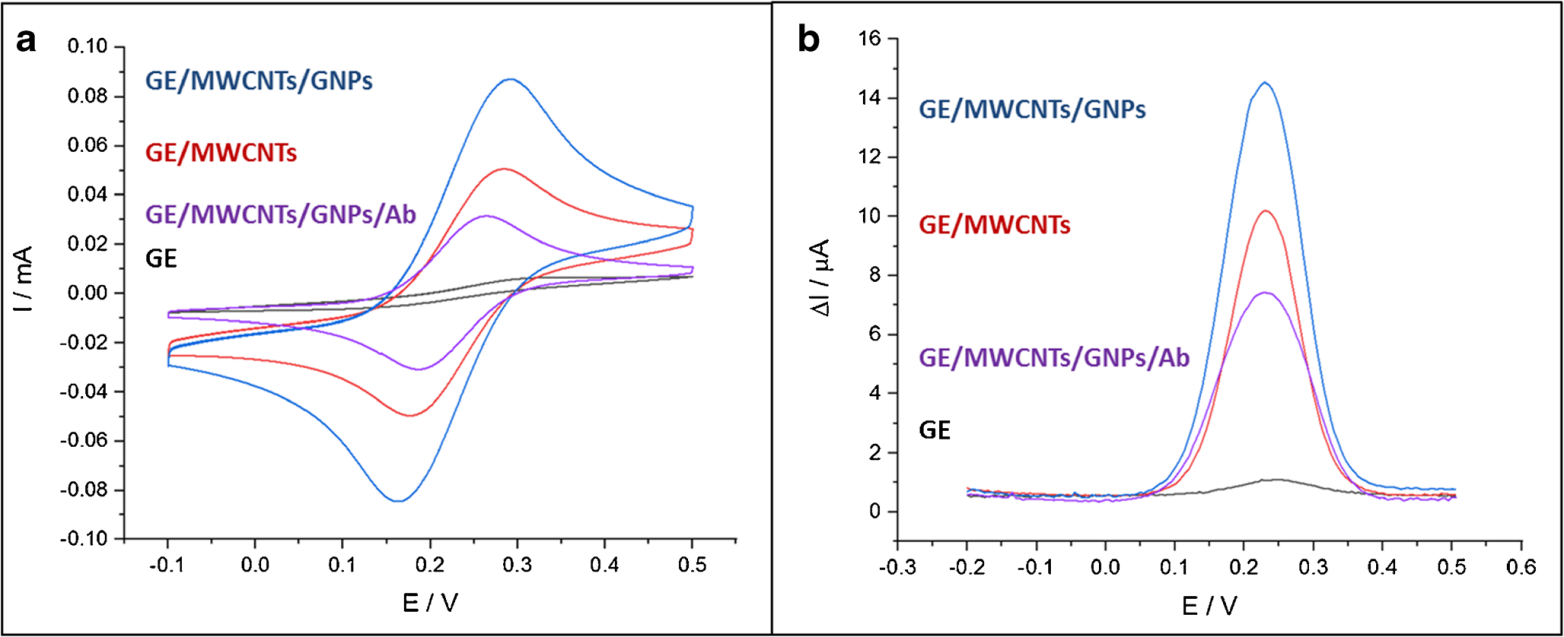
Cyclic voltammograms **a** and differential pulse voltammograms **b** performed on bare GE, GE/MWCNTs, GE/MWCNTs/GNPs, and GE/MWCNTs/GNPs/dengue antibodies in 3 mmol L^−1^ K_4_[Fe (CN)_6_]^4−^/K_3_[Fe (CN)_6_]^3−^ (1:1) in 1×PBS solution pH = 7.4. Scan rate 100 mV s^−1^

Upon recognition between the antibody and the dengue toxin, the surface of the electrode will be blocked due to the presence of biomolecules, markedly decreasing the observed Fe (II/III) redox current. Therefore, monitoring the evolution of the ferrocene-labeled probe electrochemical signals would lead to sensitive transduction of target hybridization events. The large electrode area provided by the MWCNTs/GNPs nanocomposite may significantly enhance the electrochemical signal compared to a planar sensing electrode. Also, thanks to the fast electron transfer property of the MWCNTs, the electrochemical current will no longer be limited by the electron transfer but by the diffusion of the probe through the molecular layer. This behavior was also investigated with different redox probes (see Figure 2S and Figure 3S). Furthermore, the three-dimensional (3D) network of the nanocomposite electrode promoted and facilitated the reaction between the antibody and the toxin, achieving a very low limit of detection.

This work took advantage of the recent improvements in terms of nanofabrication in order to achieve an efficient biosensor protocol that can be adapted to the detection of different biomolecules. After optimization, this proposed immunosensor achieved outstanding performance in terms of reproducibility, sensitivity, and linear range compared to the literature.

## Experimental section

### Preparation of gold electrode

Gold electrode chips were fabricated on optically polished PYREX borosilicate glass. A 100 nm layer of thermally evaporated Au on 10 nm Ti was patterned by standard UV photolithography and lift-off process. The yielded Au surface area was 0.00078 cm^2^ (diameter 0.3 mm).

### Carbon nanotube film preparation

Prior to any modification, Au electrodes were cleaned in O_2_ plasma (O_2_/N_2_) at 100 W for 5 min. Afterwards, the electrodes were rinsed in ethanol for 10 min and then rinsed 10 m in water before being dried with N_2_. Following this procedure, the GE was modified with MWCNTs according to a process presented in our previous work [15]. The first step consists of suspending MWCNTs in H_2_O (5 mg/L). The dispersion is ensured by an ultrasonic bath for 30 min. The resulting MWCNTs dispersion is then left to stand for 24 h to eliminate the aggregates of MWCNTs by sedimentation. The supernatant is then filtered through a cellulose filter (Sartorius, 0.45 μm, *Ø* = 2.5 cm) by vacuum filtration, the filtration rate was kept constant for each different filtered volume of MCNT solution. The advantage of this technique over the conventional drop-casting technique is the high reproducibility of the film obtained, as well as the ability to control the thickness and the porosity of the MWCNTs layer. Typically, for filtered volumes of 500, 300, and 100 mL, films corresponding to 4, 2.6, and 1.6 μm in thickness are obtained with a corresponding roughness of ± 0.8, 1.2, and 1.4 μm. A high roughness can be attributed to the high porosity of the materials, hence the difference of thickness at different points on the MWCNT layer. Finally, the layer of MWCNTs is transferred to the gold electrode by deposing the layer on the surface and dissolving the cellulose filter with acetone washings. After rinsing several times to remove any cellulose residue, the electrodes can be used for the electrogeneration of GNPs. This step also ensures the absence of cellulose since, in the presence of residue, the chronoamperometry used to form the nanoparticles cannot operate, the reaction being blocked by the cellulose.

### Electrodeposition of gold nanoparticles

The as-modified electrode was then used to perform the electrodeposition of GNPs onto the surface of the porous network of MWCNTs. This deposition was realized by electrochemical reduction of the precursor HAuCl_4_ (5 mmol L^−1^) in a solution of chloridric acid [11, 20]. A potential of − 0.5 V vs. Ag/AgCl/sat. electrode was imposed by chronoamperometry for 20 s. Several reduction times were tested, up to 1 min, in order to test and determine the optimal size of the nanoparticles obtained. For this work, a time of 20s offers GNP with the best amplification of the electrochemical signal (see Fig. 5S).

### Fabrication of dengue virus electrochemical sensors

The electrogenerated GNPs were firstly modified with cysteamine. The modification was performed by incubating the electrode in 100 μL of cysteamine (5 mmol L^−1^) for 1 h. The presence of a thiol functional group within this molecule allowed a spontaneous reaction with the gold surface, thereby introducing an amine end group that can be used for the immobilization of antibody [21].

The immobilization of vDENV2 antibodies was performed by using the standard EDC/NHS coupling technique. The electrode was incubated in 4 mmol L^−1^ of EDC, 10 mmol L^−1^ of NHS and 100 μL of vDENV2 antibodies (1 μgmL^−1^) overnight (16 h) at 4 °C to bind antibodies with the GNPs through amide bonds and rinsed with 1×PBS (0.01 M) solution afterwards. In order to block the unoccupied sites and reduce the non-specific adsorption, the electrode was incubated in a BSA solution (1% W/V) for 1 h [22].

Detection of RvDEN2-NS1 was then performed at different concentrations, in 1xPBS solution. The interaction was performed by drop-casting 75 μL of the solution containing different concentrations of toxin on the electrode surface and incubating for 30 min following a previously optimized procedure. The electrode was incubated with increasing concentrations of the toxin and was carefully rinsed with 1×PBS solution between each measurement. These measurements were performed on three different electrodes. The detection was carried out by differential pulse voltammetry between − 0.1 and 0.4 V vs. Ag/AgCl/sat. electrode.

### Assays in human serum

In order to further investigate the performances of the device, the biosensor was subjected to several tests under real conditions. The detection was performed in human serum spiked with different concentrations of dengue toxin. For a typical test, 50 μL of serum is used to incubate the electrode for 30 min. The electrode is then rinsed thoroughly three times with a 1×PBS solution. It is then immersed in a three electrode cell containing 3 mmol L^−1^ Fe (CN)_6_]^4−^/[Fe (CN)_6_]^3−^ in 1×PBS solution. The detection can be carried out immediately and in a few minutes by DPV.

## Results and discussion

### Characterization of the modified electrode

Before the detection of dengue toxin, the biosensor has been characterized using several methods. The system was structurally studied by SEM. Figure 2 presents images of the electrode modified by MWCNTs (Fig. 2a and b) and of the electrode after electrodeposition of GNPs (Fig. 2c and d).

Figure 2a and b show that the MWCNTs formed a porous and homogeneous structure of interconnected nanotubes. Regarding the adhesion of MWCNTs on the surface of the gold electrode, it has been shown that this interaction is mainly due to van der Waals forces [23]. In the study presented here, the formed nanotube layer exhibited a strong adhesion to the gold surface and was resistant to aqueous washing.

Figure 2c and d show the electrode after GNP electrogeneration. According to these images, the nanoparticles were uniformly distributed on the MWCNT surface during the first nucleation phase. However, during growth, the gold deposition occurred preferentially in contact with existing GNPs. This led to the formation of agglomerates of nanoparticles with a size comprised between 10 and 200 nm. This growth phase followed Oswald’s ripening theory [24, 25]. This is translated by the gradual disappearance of small nucleus and increased size of coarse nucleus over time, since it is less expensive in terms of surface energy to have a single large particle than two small particles. Energy-dispersive X-ray spectroscopy (EDX) analysis was also performed to confirm the elements composing the nanoparticles (see SI).

It is important to note that the porous nature of the MWCNTs network was preserved after electrogeneration of the GNPs. This property is essential to allow the diffusion of the redox probe and a good electron exchange with the electrode. To have a better understanding of the effect of this structure on the redox signal, further study of this material was carried out by electrochemistry.

The system was first analyzed by CV. The measurements were carried out at a scanning speed of 0.1 V/s in 1×PBS solution in the presence of 3 × 10^−3^ mol L^−1^ K_4_[Fe (CN)_6_]^4−^/K_3_[Fe (CN)_6_]^3−^ solution between −0.1 and 0.5 V vs. Ag/AgCl. The variation of the redox peak current intensities was directly related to the electron transfer and can therefore be used to collect information about the system during the construction process. The results are presented in Fig. 3a.

First, a difference can be noted between the electrochemical behavior of the bare gold electrode and the electrode modified with the MWCNTs layer. This step was accompanied by a strong increase in the current intensity of the cathodic peak, from 5.0 × 10^−6^ to 5.1 × 10^−5^ A. It translates a better electron transfer between the redox probe and the electrode due to the increased surface area provided by the MWCNTs. This behavior also confirmed to a good adhesion and contact of the MWCNTs to the surface of the gold electrode, resulting in a good conductivity between these two materials.

Subsequently, after the electrodeposition of the gold nanoparticles, a second increase in the cathodic current, from 5.1 × 10^−5^ A to 8.7 × 10^−5^ A, was observed. The spherical gold particles deposited on the walls of the MWCNTs further increase the surface area.

Finally, when antibodies were immobilized on the surface of the electrode, the redox peak current of Fe (II/III) decreased (from 8.7 × 10^−5^ A to 3.1 × 10^−5^ A). This behavior can be explained by the steric hindrance caused by the immobilized biomolecule, the antibody having a molecular weight of approximately 155 KDa. Another parameter to take into account is the charge carried by the antibody molecules. At pH 7.4, the antibody is negatively charged [26]. The electron transfer is therefore reduced by the electrostatic repulsion between the negatively charged bioreceptor and redox probe.

The DPV measurements confirmed these observations. This technique, unlike the cyclic voltammetry, focuses on the Faradaic current analysis and minimizes the charging effect of the double layer. In the presented system, the Faradaic current comes from the electron transfer between the redox probe and the electrode and is directly related to the changes at the interface. The results are presented in Fig. 3b.

Firstly, an increase in Faradaic current was observed after each modification step of the GE with MWCNTs and electrodeposited GNPs showing peak currents of 1.1, 10.2 and 14.5 μA, respectively. After immobilization of the antibodies, the current dropped to a value of 7.4 μA. These observations joined those made by CV, namely an improvement of the electron transfer due to the presence of nanometric conductive materials and a reduction of the redox probe diffusion caused by the presence of the bioreceptor. The immobilization of the antibodies clearly led to the formation of an electron transfer and mass transfer blocking layer.

**Fig. 4:**
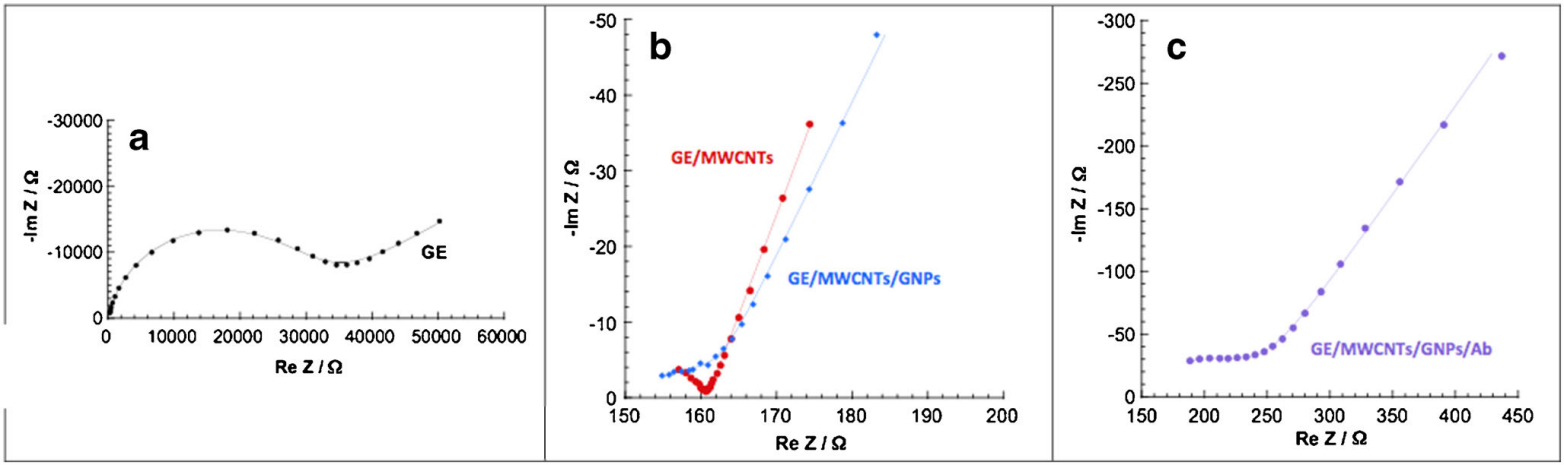
Nyquist plots of impedance spectra performed at 0.22 V vs. Ag/AgCl using Fe (CN)_6_^3−^/(CN)_6_^4−^ (3 × 10^−3^ mol L^−1^) in 0.1 mol L^−1^ PBS solution, pH = 7.4 on **a** GE, **b** GE/MWCNTs in red and GE/MWCNTs /GNPs in blue, and **c** GE/MWCNTs/GNPs/Ab

Construction of the molecular edifice was also followed by electrochemical impedance spectroscopy. The measurements were performed at 0.2 V vs Ag/AgCl (Sat. KCl), in a solution of 3 mmol L^−1^ K_4_[Fe (CN)_6_]^4−^/K_3_[Fe (CN)_6_]^3−^ (1:1) in 1**×**PBS solution. A frequency range from 200 kHz to 200 mHz was applied with an amplitude of 10 mV. This technique allowed a better understanding of the system and the phenomena taking place at the interface by transposing the system into an equivalent electrical circuit. For this kind of system, a classical Randles circuit is usually chosen. This model consists of the ohmic resistance of the electrolyte solution and the modified electrode, the charge transfer resistance in series with a Warburg element and in parallel with a double layer capacity [27, 28]. The resulting Nyquist diagrams for the bare gold electrode and the modified electrodes are presented in Fig. 4.

In this representation, the first semicircle is attributed to the charge transfer resistance in parallel of double layer capacitance. The second part, at low frequency, represents the contribution of the Warburg impedance.

It may be noted that the homemade gold electrode had a classic response to the redox probe [29]. A well-defined charge transfer resistance is present followed by the contribution due to diffusion process at lower frequency. Since the electrode was not yet modified, the reaction was mainly limited by the electron transfer and not by the diffusion of the redox probe.

Once the electrode had been modified by the MWCNTs film (Fig. 4b), a large decrease in the charge transfer resistance was observed, denoted by the disappearance of the first semicircle previously visible at high frequency. The introduction of MWCNTs therefore entailed a significant change in the electrochemical response of the system. Thanks to the fast electron transfer offered by the MWCNTs, the reaction is now limited by the diffusion. As shown by Fig. 4b, the charge transfer resistance became negligible before the diffusion resistance. The same phenomenon was observed after electrogeneration of GNPs. After the immobilization of dengue antibodies, the charge transfer resistance increased due to steric hindrance caused by the presence of numerous antibodies on the surface. The fitting of the data confirmed those observations and gave valuable information about the reaction at the interface (Table 1). It clearly demonstrated the major contribution of the MWCNTs and GNPs regarding the improvement of the electron transfer and the electroactive surface area of the electrode. If it is assumed that the capacity remained of the same type whatever the configuration, it can be said that the ratio of the double layers capacity corresponds to the ratio of the electrochemically active surfaces. Thus between GE and GE/MWCNTs, the electrochemically active area increased by a factor of thirty, and by a factor of ten between GE/MWCNTs and GE/MWCNTs/GNPs. These values were only given by orders of magnitude because the uncertainties of the fitting were very high for the GE/MWCNTs, and GE/MWCNTs/GNPs configurations. These uncertainties were caused by the low values of the measured impedances on those electrodes.

**Table 1:** Values of equivalent circuit elements (*R*_CT_,*C*_DL_) obtained after fitting of the EIS experimental data corresponding to the changes in impedance on the gold electrode (GE), after deposition of MWCNTs (GE/MWCNTs), after nanoparticles electrogeneration (GE/MWCNTs/GNPs) and after antibodies immobilization (GE/MWCNTs/GNPs/Ab)

	GE	GE/MWCNTs	GE/MWCNTs/GNPs	GE/MWCNTs/GNPs/Ab
*R*_CT_/*Ω*	27,800 ± 800	20 ± 4	13 ± 5	104 ± 9
*C*_DL_/*F*	(3.3 ± 0.1) × 10^−8^	(1.0 ± 0.2) × 10^−6^	(1.0 ± 0.4) × 10^−5^	(1.3 ± 0.1) × 10^−7^

### Detection of dengue toxin

For monitoring the concentration of the dengue toxin, DPV was chosen over CV. The main advantages with pulse techniques are the difference in decay rates of Faradaic and non-Faradaic current. In DPV, the charging current of the double layer capacitance is negligible thanks to the differential measurement. The short pulse time also increases the measured currents. As a result, the obtained differential pulse voltammogram exhibits an increased ratio of Faradaic to non-Faradaic current, allowing better discrimination against background processes. Since the intensity of the peak current obtained is directly related to the amount of toxin immobilized on the surface of the electrode, small changes at the interface and a small amount of targets can be detected.

Figure 4 shows the DPV results obtained after the incubation of the electrode with different RvDEN2-NS1 concentrations between 1 × 10^−12^ and 1 × 10^−6^ g mL^−1^. Between each concentration, the electrode was carefully rinsed with a PBS solution, and these results were repeated with three different electrodes.

**Fig. 5:**
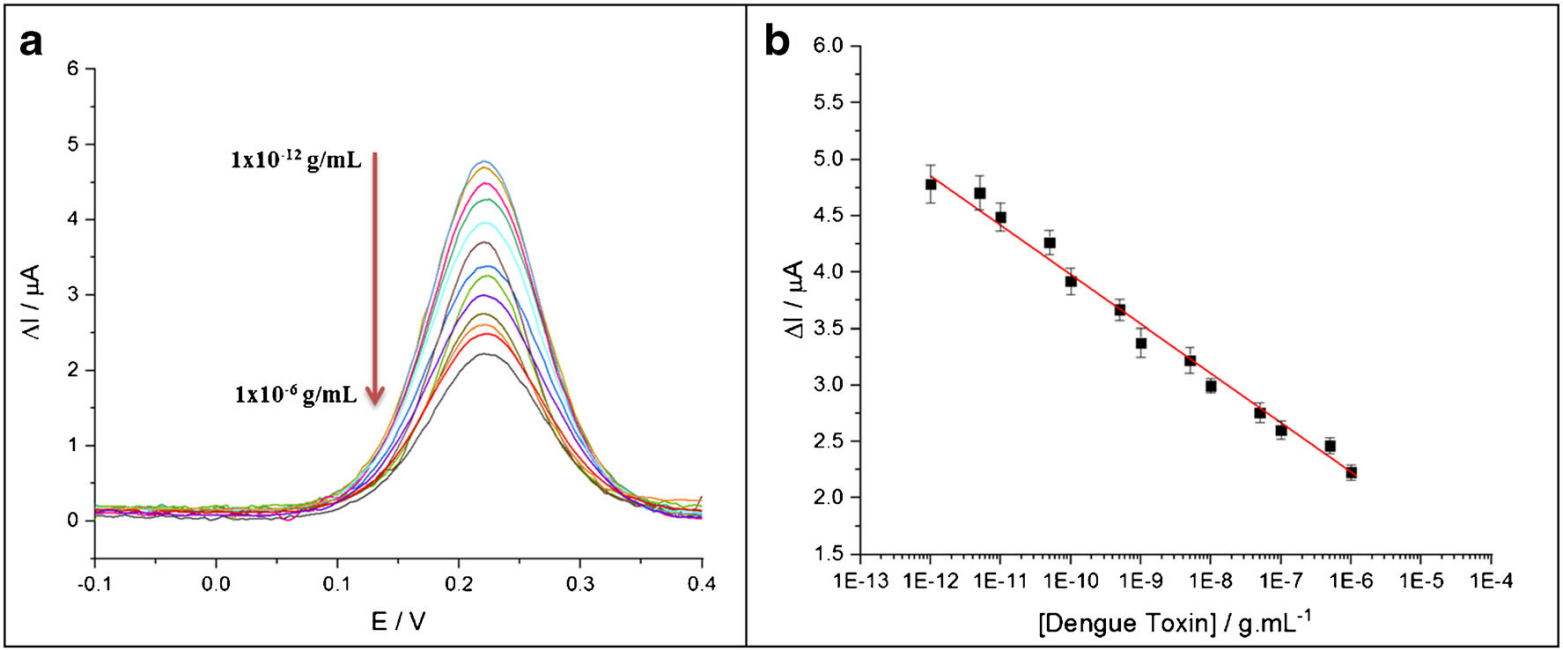
**a** DPV curves after incubating with various concentrations of the dengue toxin in 1**×**PBS solution, pH = 7.4. From top to bottom: 0.001, 0.005, 0.01, 0.05, 0.1, 0.5, 1, 5, 10, 50, 100, 500 and 1000 ng mL^−1^. **b** Calibration plot for the biosensor corresponding to the changes in current intensity upon detection of dengue toxin at different concentrations in buffer. Experimental (dots) and linear regression (lines) are presented. Linear regression: slope = − 0.437 ± 0.01 μA per order of magnitude; *R*^2^ = 0.985

As it can be seen in Fig. 5a, the intensity of the Faradaic current decreased when toxin was added. The recognition event seemed to greatly inhibit the reaction at the interface. A total decrease from 4.78 to 2.22 μA was observed between 1 × 10^−12^ and 1 × 10^−6^ g mL^−1^. This was consistent with the expected behavior that an increasing RvDEN2-NS1 presence densified the protein layer leading to reduced diffusion of the probe. Moreover, the isoelectric point of this biomolecule is 5.7 [30] and it is therefore negatively charged at pH 7.4. This charge will increase the repulsion already observed after antibody immobilization. As a result, less redox probes were detected on the electrode which can explain the decrease of the signal.

Figure 5b shows the current change as a function of toxin concentration. This variation follows a semi-logarithmic evolution between 1 × 10^−12^ and 1 × 10^−6^ g/mL of toxin. The sensitivity observed for this concentration range was − 0.437 ± 0.01 μA per decade with a correlation factor (*R*^2^) of 0.985. For this system, the theoretical limit of detection (LOD) obtained after taking into account the standard deviation and the signal/background ratio (three times the signal-to-background ratio, 3S/N) is 3 × 10^−13^ g/mL. However, this concentration could not be discriminated from the background when tested experimentally. After assessing different concentrations between 3 × 10^−13^ g/mL and 1 × 10^−12^ g/mL, the experimental LOD was found to be 1 × 10^−12^ g/mL.

Usually, this type of biological system is described by the Langmuir isotherm, where the obtained current is linearly correlated to the target concentrations. However, this model is simplified by not taking into account parameters such as the inhomogeneity of the surface or the heterogeneity of the adsorption sites. Nonetheless, for biosystems, the recognition event between antigen and antibody on a specific adsorption site directly affects the adjacent sites. This phenomenon is described by the Langmuir-Freundlich isotherm which predicts that the recognition event firstly occurs on the most accessible domains [31]. The Temkin isotherm deepened this model by considering an inhomogeneous surface, where the adsorption sites are initially not equivalent. This model was firstly used to describe a heterogeneous protein adsorption [32]. According to the experimental data, and after using these three different models, the Temkin isotherm is best suited for the presented system.

Table 1s lists the performance of different biosensors for the detection of dengue toxin available in the literature. The comparison shows that the works presented in this paper offer exceptional results in terms of detection limit and sensitivity. The device covers 6 orders of magnitude, between 0.001 and 1000 ng mL^−1^ and achieves a very low detection limit of 1 × 10^−12^ g/mL which is two orders of magnitude lower than the average reported in literature (Table 1S). Such improvement in LOD represents the appropriateness of the proposed biosensor to detect dengue infection in an early stage of the disease [33, 34].

This improvement comes mainly from the very large surface area offered by the MWCNTs/GNPs nanocomposite. As shown previously, this increase in the electrode surface led to much higher currents than those obtained for a conventional electrode. By increasing the signal-to-background ratio and by using DPV as a very sensitive technique, it was possible to detect small amounts of analyte. The large surface also enabled to immobilize a large quantity of bioreceptor, thus increasing the sensitivity (− 0.437 ± 0.01 μA per decade) of the biosensor. The second major improvement comes from the fast electron transfer offered by the MWCNTs/GNPs nanocomposite that leads to a diffusion-limited system. Since the activity of immobilized antibodies depends on their orientation, the three-dimensional structure of the porous network of MWCNTs and GNPs provided improved accessibility of the analyte and improved the diffusion. This promoted and facilitated the reaction between the antibody and the toxin, allowing to record very small changes in concentration and therefore to achieve a very low limit of detection. The benefit of the 3D-type diffusion is well described in the diffusion-reaction model [35].

### Stability and selectivity tests

**Fig. 6:**
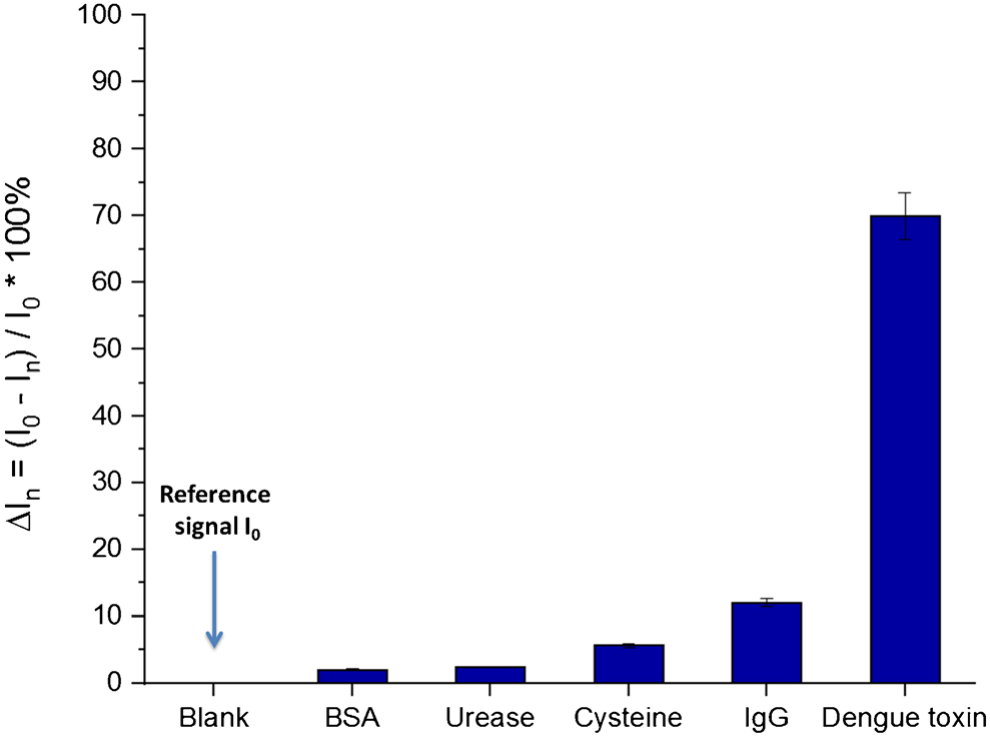
The difference in current intensities after incubation of the antigen-modified electrodes with different biomolecules: bovine serum albumin (BSA), urease, cysteine, rabies antibodies (IgG), and the specific dengue toxin

The selectivity of the system towards the detection of dengue toxin was investigated by exposing the biosensor to different non-specific targets. The dengue antibody-modified gold electrodes were incubated with solutions containing non-specific bovine serum albumin, urease, cysteine, rabies antibodies (IgG), and specific dengue toxin. The concentration (1 μg mL^−1^ in 1**×**PBS solution) and the incubation time (30 min) were kept constant for all experiments. The results of this study are presented in Fig. 6. The measurements were carried out using DPV in 3 mmol L^−1^ Fe (II/III). Figure 6 shows the relative current intensity variation, Δ*I*_*n*_ = (*I*_0_ − *I*_*n*_)/*I*_0_ × 100%), after incubation with the different interferers. *I*_0_ is the reference signal obtained after dengue antibodies immobilization and *I*_*n*_ is the signal obtained after incubation.

**Fig. 7:**
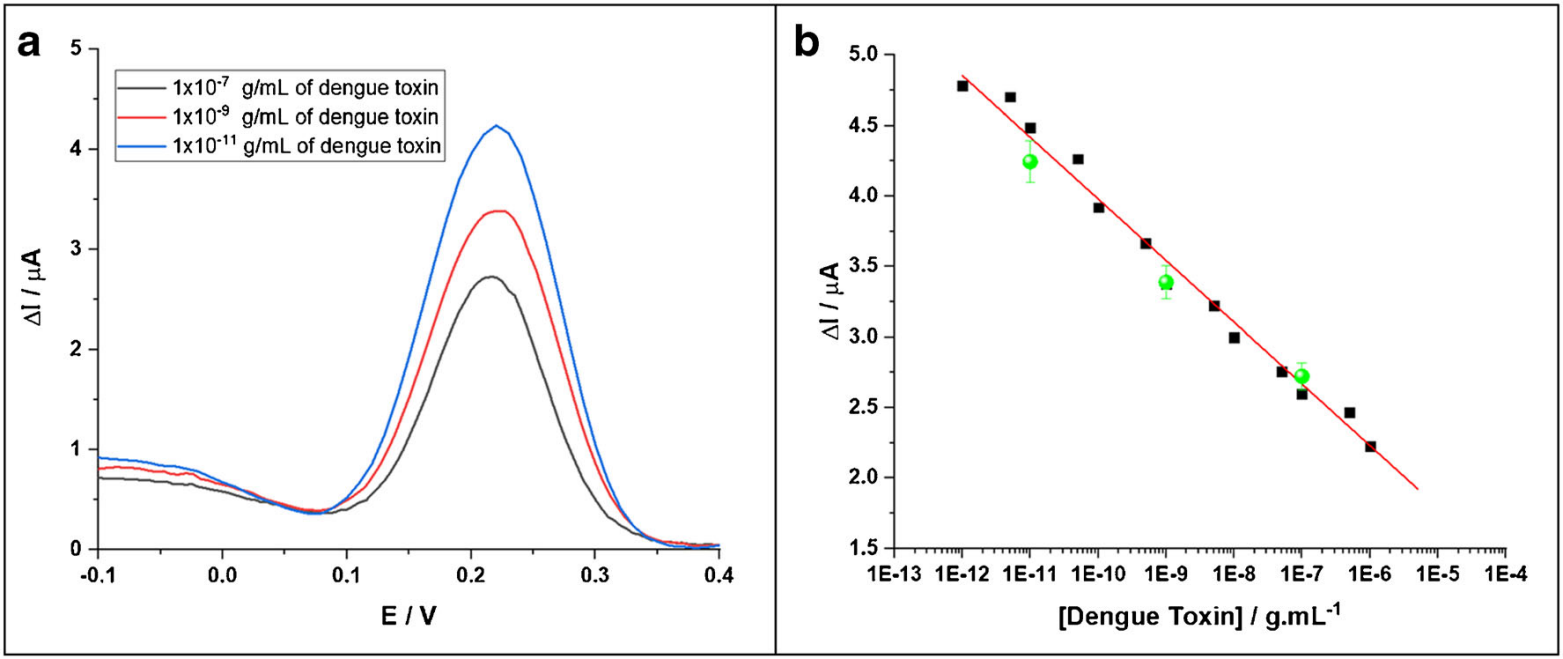
**a** DPV curves after incubating with various concentrations of the dengue toxin in human serum. From top to bottom: 0.01, 1, 100 ng mL^−1^. **b** Calibration plot for the biosensor corresponding to the changes in current intensity upon detection of dengue toxin. The experimental data (dots) for the tests in human serum are also presented

From this study, it can be seen that the system had no significant response towards non-specific targets. The incubation of the different biomolecule just resulted in a very small change in the current intensity compared to the initial current. The strongest non-specific adsorption occurred after exposing the IgG system, leading to 12% of current intensity reduction compared to the original signal. After incubation with the specific dengue toxin a 70% reduction of the blank current intensity was observed. This clear decrease was attributed to the specificity of the biosensor to dengue toxin. The individual voltammograms of the different non-specific targets can be found in Fig. 7S for more information.

The stability of the biosensor was also tested as shown in Fig. 6S. This parameter is very important in electrochemistry since it validates the results observed and eliminates any false positives caused by a possible drift of the system. The proposed biosensor exhibited a stable signal after more than 10 consecutive measurements in the buffer, which ensured the validity of the response observed during the detection of RvDEN2-NS1.

### Detection of dengue toxin in human serum

As described above, tests were carried out in human serum. Three different concentrations were analyzed and the results were compared to the calibration line previously established. The experimental data are presented below Fig. 7.

Three toxin concentrations were tested with several electrodes in human serum. The data show that the redox peak current follows the calibration plot drawn from the detection performed in PBS, taking into account the standard deviation. According to data found in the literature, the concentration range required for detection of dengue NS1 from human serum sample is comprised between 0.001 and 2 μg/mL in human serum [33, 34]. This shows the feasibility and the interest of the proposed system with regard to the detection of the dengue toxin in real samples. In addition, detection is very simple and quick to perform, ideal for a point-of-care device. Assays can also be performed at a single potential for easier integration (0.22 V).

## Conclusion

The presented work highlights the realization of an electrochemical biosensor for the detection of dengue toxin. This sensor was based on the modification of a gold electrode with a nanocomposite that took advantage of the properties of MWCNTs and GNPs. The resulting nanostructured electrode improved the electron transfer between the redox probe and the electrode surface, thus inducing important enhancement of the electrochemical signal. The 3D structure also facilitated the recognition event between the target and the bioreceptor, allowing the monitoring of very small concentration of dengue toxin. The proposed electrochemical biosensor exhibited a wide linear range and low detection limit altogether with high sensitivity. Assays carried out in human serum underline the interest of the device for rapid diagnostics in the medical environment. Regarding stability, the choice of the redox probe and the use of nanomaterials can greatly influence this parameter and must be taken into account. Finally, the interest of this work is also based on the versatility of the system since the used immobilization technique can be appropriate for other biomolecules and represents an efficient platform for other biological targets of interest.

## Electronic supplementary material

ESM 1 (DOCX 841 kb).
