# Supplementary material for: Voltammetric sensing of recombinant viral dengue virus 2 NS1 based on Au nanoparticle–decorated multiwalled carbon nanotube composites
Source: Mikrochim Acta. 2020 Jun 2;187(6):363. doi: 10.1007/s00604-020-04339-y (PMC7266806; doi:10.1007/s00604-020-04339-y)
Supplement: Supplementary file 1 — (DOCX 841 kb). [file 604_2020_4339_MOESM1_ESM.docx]

**Electronic Supporting Material on the Microchimica Acta publication entitled**

Voltammetric sensing of recombinant viral dengue virus 2 NS1 based on Au nanoparticle-decorated multiwalled carbon nanotube composites

Quentin Palomar, ^a^ XingXing Xu, ^a^ Chantal Gondran, ^b^ Michael Holzinger, ^b^ Serge Cosnier ^b^ and Zhen Zhang, ^a^^[[1]](#footnote-1)^

^a^ Division of Solid-State Electronics, Department of Engineering Sciences, The Ångström Laboratory, Uppsala University, P.O. Box 534, SE-751 21 Uppsala, Sweden

^b^ Univ. Grenoble Alpes, CNRS, DCM UMR 5250, F 38000, Grenoble, France

1. ***Chemicals and Materials:***

Gold(III) chloride hydrate (HAuCL_4_), cysteamine (NH_2_CH_2_CH_2_SH), Hydrofluoric acid (HF, 40%), Bovine Serum Albumin (98%), Urease from Canavalia ensiformis, L-Cysteine (97%), Anti-Rabies antibody, Phosphate buffered saline (PBS) tablets, N-Hydroxysuccinimide (NHS, 98%), N-(3-Dimethylaminopropyl)-N′-ethylcarbodiimide hydrochloride (EDC, 98%), Potassium hexacyanoferrate(III) (K_3_Fe(CN)_6_, 99.5%) and Potassium hexacyanoferrate(II) (K_4_Fe(CN)_6_, 99.5%) were all purchased from Merck-Sigma. Commercial grade thin multi-walled carbon nanotubes (9.5 nm diameter, purity >95%) were obtained from Nanocyl and used as received without any purification step. Recombinant viral dengue virus 2 NS1 (RvDEN2-NS1, 95%) and viral dengue virus 2 NS1 antibody (vDENV2 Mab, 95%) were purchased from bio-techne, USA. All chemicals were used as received without any further purification. Water was supplied by nanopure water system.

1. ***Instruments and Characterization:***

All electrochemical measurements were carried out using a conventional three-electrode cell and a VSP 300 (Bio-Logic, France) electrochemical workstation. Modified homemade gold electrodes (Ø = 0.3 mm) were used as working electrode. A platinum wire (diameter 0.5 mm, ALS Co., Japan) and a saturated silver chloride electrode (Ag/AgCl/sat.) served as counter and reference electrodes, respectively. All potentials were measured and referred to the Ag/AgCl/sat electrode. Saline phosphate buffer at a concentration of 0.1 molL^-1^ (pH 7.4) was employed as the supporting electrolyte unless specifically indicated and a solution of 3 mmolL^-1^ K_3_Fe(CN)_6_/K_4_Fe(CN)_6_ was used as redox probe. Each stage of the biosensor construction was followed by cyclic voltammetry and electrochemical impedance spectroscopy measurements. The detection of dengue toxin was carried out by differential pulse voltammetry. The data was recorded and processed using EC lab software.

O_2_ plasma was performed in a Tepla 300 plasma processor (PVA TePla, Germany).

Scanning electron microscopy (SEM) images were captured with Zeiss 6 LEO 1530 (Germany).

1. ***Energy-dispersive X-ray spectroscopy Characterization:***

To confirm the presence of GNP after electrogeneration, Energy-dispersive X-ray spectroscopy (EDX) analysis has been performed on the gold electrode modified with CNTs and on gold electrode modified with CNTs/GNPs. The results are shown below (Figure 1S).


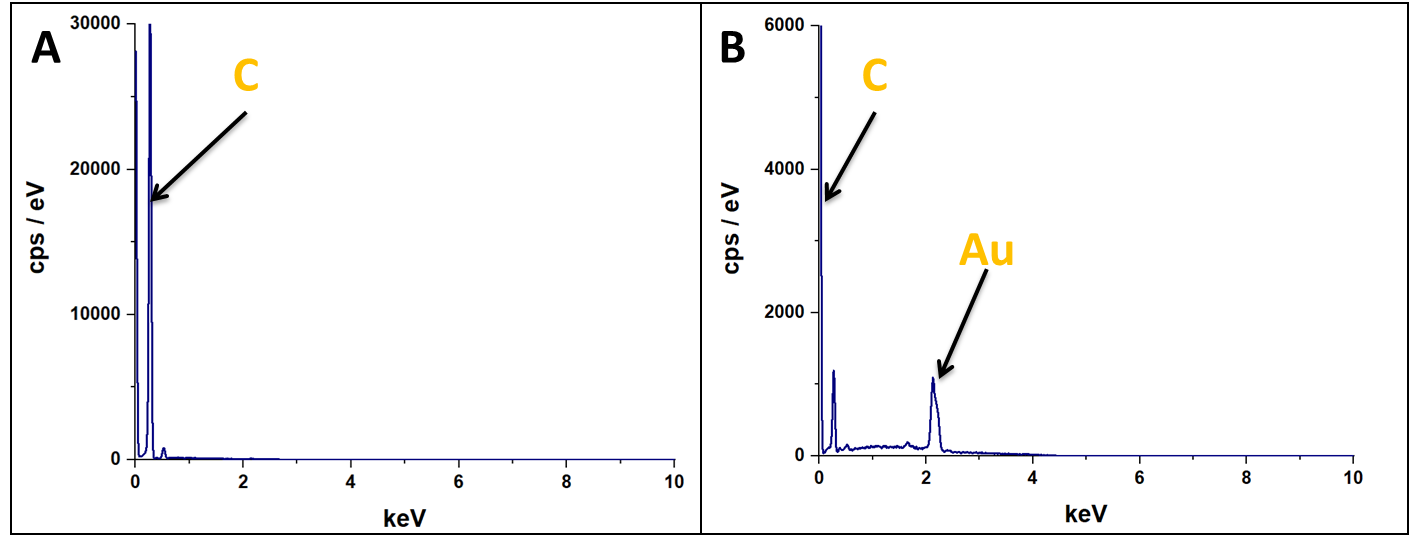


Figure 1S: Energy-dispersive X-ray spectroscopy (EDX) analysis of the A/ gold electrode modified with the CNT layer and B/ the gold electrode modified with CNTs/GNPs.

The first analysis confirmed the presence of carbon mainly and especially the absence of gold. There were also traces of oxygen due to the oxygen functions present on the surface of the CNTs. The second analysis proved the formation of GNPs on the surface of CNTs as a clear peak appears around 2 KeV corresponding to Au element.

1. ***Characterization with different redox probes:***

To have a better understanding of the impact of MWCNTs on electron transfer, their contribution was analyzed using two other electrochemical probes, namely hydroquinone and hexaamine ruthenium redox couple (Ru(NH_3_)_6_^2+^/ Ru(NH_3_)_6_^3+^).

Figure 2S shows the Nyquist plots of impedance spectra performed on the bare gold electrode and on the electrode modified with MWCNTs in 3 mM of hydroquinone in 1xPBS solution.

**
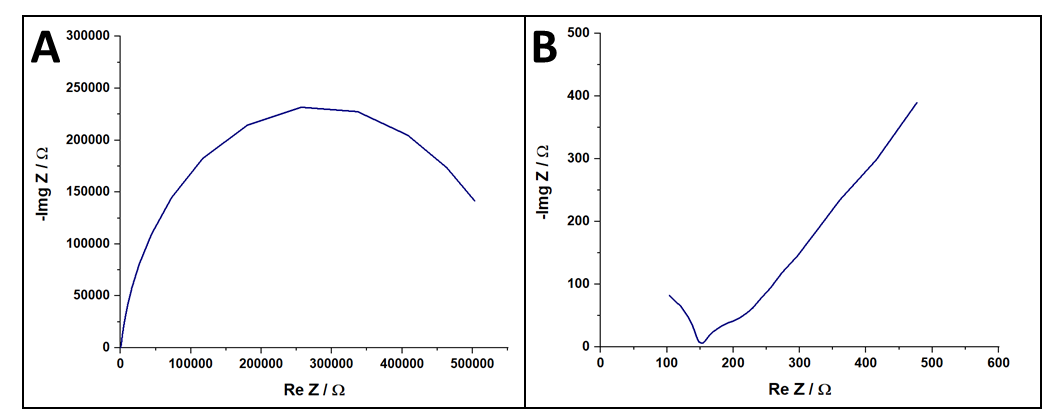
**

Figure 2S: Nyquist plots of impedance spectra performed at 0.1V vs. Ag/AgCl using hydroquinone (3×10^-3^ mol L^-1^) in 1x PBS solution, pH = 7.4 on (A) GE , (B) GE/MWCNTs.

The first Nyquist plot (Figure 2S-A) reflects a strong electron transfer resistance, characterized by the presence of a large semi-circle at high frequency corresponding to R_CT_. This high resistance is due to the fact that hydroquinone is not a good probe for gold electrode and can form a blocking layer by adsorption on the surface^1^. After modification by the layer of MWCNTs, a very strong decrease in the overall resistance of the system is observed as well as a disappearance of the semicircle. As in the case of Fe(CN)_6_]^4−^/ [Fe(CN)_6_]^3−^, adding MWCNTs greatly modified the electrochemical signal by increasing the surface and accelerating the electronic transfer, leading to a diffusion controlled process.

The same tests were performed in the presence of 3 mM Ru(NH_3_)_6_^2+^/ Ru(NH_3_)_6_^3+^. Figure 3S-A exhibits the Nyquist plots of impedance spectra performed at -0.2V vs. Ag/AgCl on the gold electrode and the electrode modified with MWCNTs and 3S-B the corresponding DPV.

**
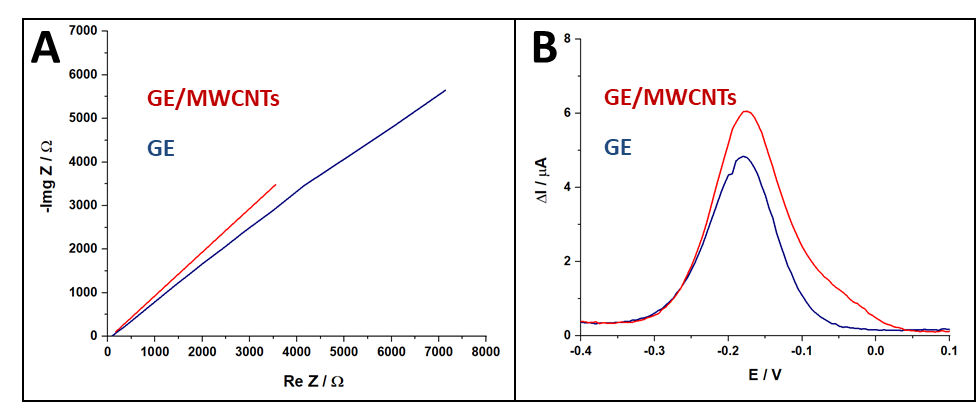
**

Figure 3S: A/ Nyquist plots of impedance spectra performed at -0.2V vs. Ag/AgCl using Ru(NH_3_)_6_^2+^/ Ru(NH_3_)_6_^3+^. (3×10^-3^ mol L^-1^) in 1xPBS solution, pH = 7.4 on GE and GE/MWCNTs. B/ differential pulse voltammograms performed on bare GE, GE/MWCNTs in 3 mmolL^-1^ Ru(NH_3_)_6_^2+^/ Ru(NH_3_)_6_^3+^ (1:1) in 1xPBS solution pH = 7.4.

Unlike that for hydroquinone, the electrochemical impedance using Ru(NH_3_)_6_^2+^/ Ru(NH_3_)_6_^3+^ presents little difference between the bare GE and the MWCNTS-modified electrode. This may be due to the fact that the electron transfer is already very good before the modification, which explains the appearance of the Nyquist plots obtained and the absence of clear semi-circle at high frequency. This observation is confirmed by the DPV, where a strong peak current is observed for the bare electrode, with a slight increase in the signal after modification by the MWCNTs. This redox couple therefore seems to be a good candidate to be used as a probe with gold electrode. However, with our system, it suffers from a lack of stability over time after several electrochemical cycles, as illustrated in Figure 4S. After immobilization of the antibodies, a semicircle appears at high frequency as the antibodies created a blocking layer. However, during consecutive scans in the presence of Ru(NH_3_)_6_^2+^/ Ru(NH_3_)_6_^3+^, an increase in resistance appears without any modification of the system. This drift can result from the degradation of the redox couple or from an adsorption at the surface of the electrode. Indeed, the dengue antibodies are negatively charged at this pH, whereas the ruthenium couple is charged positively. It can therefore form an electrostatic attraction between these two entities.

This is why the Fe(CN)_6_]^4−^/ [Fe(CN)_6_]^3−^ couple was preferred for the rest of the work.


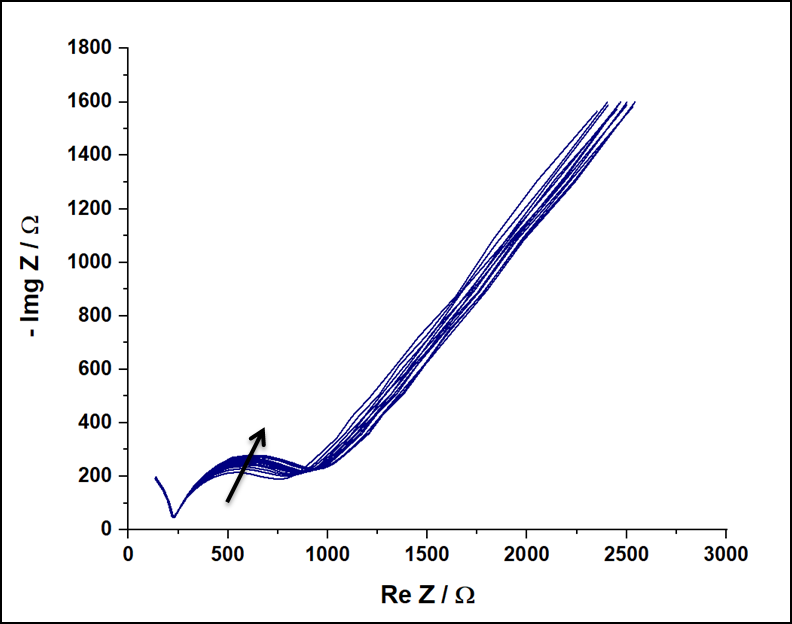


Figure 4S: Consecutive impedance scan performed at -0.2V vs. Ag/AgCl using Ru(NH_3_)_6_^2+^/ Ru(NH_3_)_6_^3+^. (3×10^-3^ mol L^-1^) in 1xPBS solution, pH = 7.4 on GE/MWCNTs/Ab.

1. ***Optimization of the GNPs electrogeneration:***

In order to obtain a size of nanoparticles in adequacy with the needs of the device, several deposition times were tested. The MWCNTs-modified electrodes were exposed to a potential of -0.5 V vs. Ag/AgCl/sat. electrode for 10 to 60s in a solution of HAuCl_4_ (5 mmolL^-1^).

Following the GNPs deposition, the electrodes were modified with dengue antibodies according to the procedure presented before and backfilled with BSA. Their electrochemical response to the redox ferri/ferrocyanide (Fe (II/III)) couple was then investigated by DPV. The results are presented in Figure 5S.


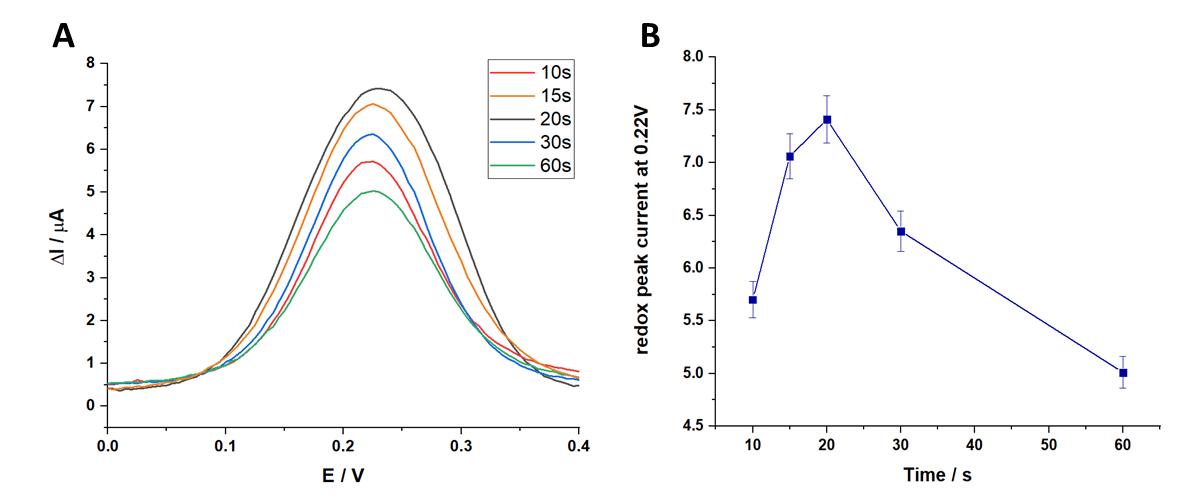


Figure 5S: A / differential pulse voltammograms performed on GE / MWCNTs / GNPs / Dengue antibodies after different electrogeneration times (10 to 60s) in 3 mmolL^-1^ K_4_[Fe(CN)_6_]^4−^/K_3_[Fe(CN)_6_]^3−^ (1:1) in 1xPBS solution pH = 7.4. B / Variation of the intensity of the redox peak current at 0.22V as a function of the deposition time of the nanoparticles.

As shown in Figure 5S, the formation of nanoparticles first increased the intensity of the current recorded before decreasing it with a peak of intensity for 20 s of GNPs electrogeneration. These data confirm the observations made previously, namely that the formation of GNPs follows the Oswald's ripening theory, where the system will tend to form bigger nanoparticles once the saturation threshold is reached. In the proposed system, it would seem that the optimum deposition time is 20s. Beyond this value, the GNPs formed become too large and there can be formation of contact grains between two nanoparticles. This presents two disadvantages. On the one hand the aggregation of two GNPs will reduce the volume / surface ratio, decreasing the specific surface area, and on the other hand, too large particles will block the pores of the MWCNTs network and thus prevent the diffusion of the redox probe, which explains the signal loss observed for generation times longer than 20s.

1. ***Characterization of the electrode stability:***

The stability of the proposed biosensor had also been tested. It is crucial to study the stability of the system with respect to different parameters such as storage, incubation time or exposure to the measurement medium. This kind of experiments will ensure that the observed response during the detection is due to the recognition event and not to uncontrolled side effects.

For this study, the sensor was firstly exposed to different incubation times in 3 mmol L^-1^ Fe (II/III) solution prepared in 1xPBS solution. The measurement was done by DPV.

For the second stability test, the electrodes were kept in a 1xPBS solution for several weeks and the remaining signal was recorded every day. Results are shown in Figure 6S. This study was conducted on 2 different sets of electrodes.


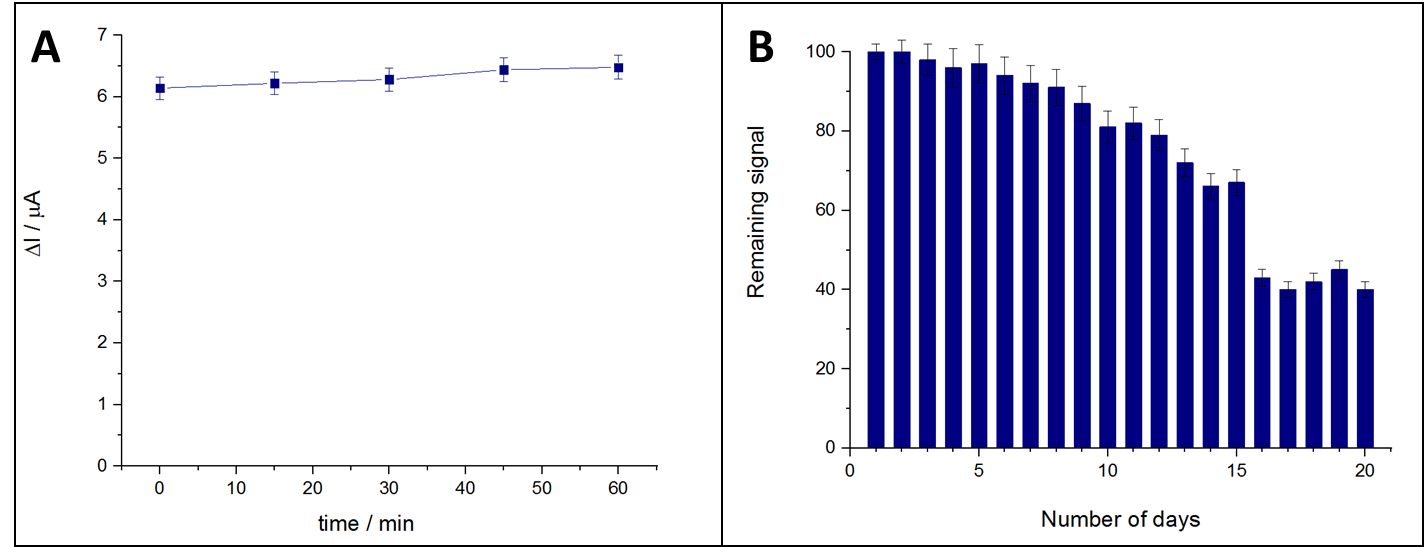


Figure 6S: Stability study after incubating the as-modified electrodes in 3 mmol L^-1^ Fe (II/III) solution prepared in 1xPBS solution for different period of time (A) and after storage in PBS solution at 4°C (B).

The system showed very good stability after different incubation times with a solution of 3 mmol L^-1^ Fe (II/III) in 1xPBS solution. A small increase in the current intensity from 6.14 to 6.48 µA was observed. Regarding the storage stability in PBS solution, the system lost 20% of the original signal after 10 days storage. This is consistent with the theoretical lifetime of an immunosensor ^2,3^.

These results attested the stability of the molecular architecture under those conditions and validated the results obtained during the detection of dengue toxin.

The electrodes were also subjected to successive DPV to study their stability after electrochemical measurements. This important parameter, often forgotten in the field, has been highlighted by Jolly et al ^4^. They have shown that a simple succession of electrochemical measurements can lead to false positive. Nevertheless, the proposed system was stable after more than 10 consecutive measurements, which ensured the validity of the response observed during the detection of RvDEN2-NS1.

1. ***Comparison with the literature:***

In order to compare the performance of the biosensor proposed in this work and those reported in literature towards the detection of the dengue virus, representative studies of several research teams are presented in Table 1S. This comparison was based on various criteria such as the used nanomaterials of the detection system, the limit of detection (LOD), or the concentration range for which the biosensor is relevant.

Table 1S: Comparison of the obtained characteristics (system used, LOD, and linearity) of different nanostructured electrochemical dengue virus biosensors.

|  | **System used** | **LOD/ ng/mL** | **Linearity/ ng/mL** |
| --- | --- | --- | --- |
| This work | GE/MWCNTs/GNPs | 0.001 | 0.001-1000 |
| Cecchetto et al^5^ | Gold electrode + mixed thiol-SAM structures | 3 | 10-1000 |
| Cavalcanti et al.^6^ | Gold electrode + recordable compact disk | 0.33 | 1-100 |
| Dias et al^7^ | Carbon nanotube-ink printed electrode | 12 | 40-2000 |
| Santos et al.^8^ | Gold electrode + SAM | 0.340 | 1-5000 |
| Sinawang et al. ^9^ | Electrochemical lateral flow immunosensor/screen printed gold electrode | 0.5 | 1-25 |
| Parkash et al.^10^ | Streptavidin/biotin system on screen printed carbon electrodes | 30 | 500-2000 |
| Nawaz et al.^11^ | Screen printed electrode + BSA/NBu_4_BF_4_ | 0.3 | 1-200 |
| Lim et al.^12^ | Gold electrode + peptide | 25 | 25-3000 |
| Dutra et al.^13^ | Carbon Ink Screen-Printed Gold Nanoparticles | 30 | 100-200 |
| Silva et al.^14^ | Poly(allylamine) sandwiched CNTs | 35 | 100-2500 |

The data reported in this table show that the presented work reveals best biosensor performances in terms of LOD and linearity range. The device allowed a detection covering 6 orders of magnitude, between 0.001 and 1000 ng mL^-1^. However, it should be noted that analyte saturation was more rapidly reached in our setup than for other systems such as Santos et al. (5000 ng mL^-1^) ^8^. This may be related to the smaller electrodes (Ø 0.3 mm) used in these works contrary to more conventional electrode size (Ø 1 mm). Navakul et al.^14^ have also developed a fast and sensitive electrochemical sensor based on GE coated with graphene oxide reinforced polymer. They achieved a limit of detection of 0.12 pfu/mL which allowed them to detect dengue fever in early stage of the disease Nevertheless, the greatest advance of the presented system was the very low detection limit value of 1×10^-12^ g/mL which was two orders of magnitude lower that the average reported in literature (table 1) and its ease of use. Such improvement in LOD represents for the proposed biosensor its appropriateness to detect dengue infection in an early stage of the disease ^15,16^.

1. ***Characterization of the biosensor specificity:***

Individual voltammograms obtained after incubation of the electrodes with non-specific targets are presented below (Figure 7S). The reference voltammogram obtained after exposition to 1.10-6 g/mL of dengue toxin is also presented. The corresponding variation in term of current intensity can be found in figure 6.


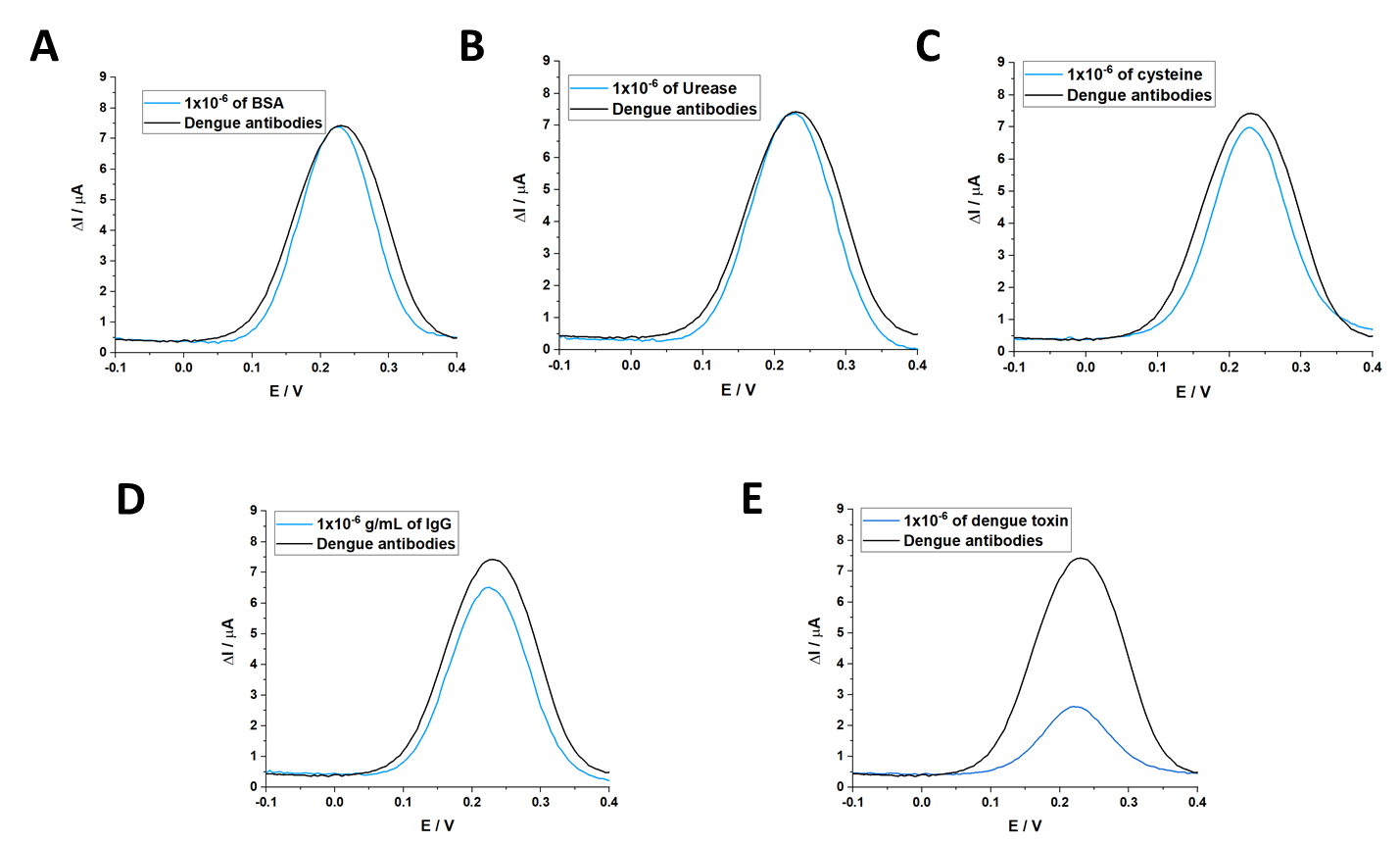


Figure 7S: / DPV curves after incubating the electrodes with various biomolecules at 1.10^-6^ g.mL in 1x PBS solution: A/ bovine serum albumin (BSA), B/ urease, C/ cysteine, D/ rabies antibodies (IgG) and E/ the specific dengue toxin.

1. ***Gold electrode picture:***

Photo showing the geometry and dimensions of the homemade electrodes before modification.


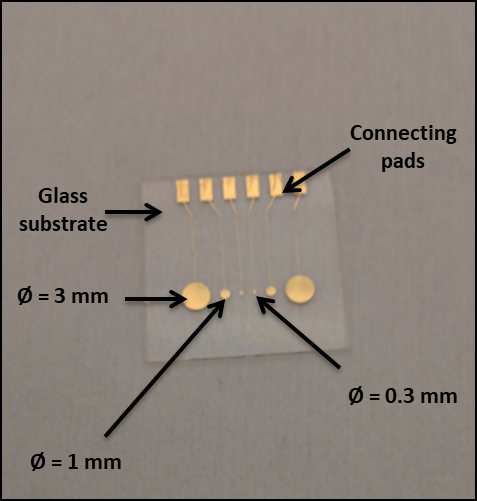


Figure 8S: picture of the homemade electrodes before modification showing the geometry and the dimension of the different electrodes.

(1) Naito, K.; Maeda, Y.; Yasui, T.; Takada, K.; Yuchi, A. Adsorption of Hydroquinone to Polycrystalline Gold Electrode and Its Effect on the Electrochemical Properties. *J. Electrochem. Soc.* **2017**, *164* (9), H670–H674. https://doi.org/10.1149/2.1641709jes.

(2) Ojeda, I.; López-Montero, J.; Moreno-Guzmán, M.; Janegitz, B. C.; González-Cortés, A.; Yáñez-Sedeño, P.; Pingarrón, J. M. Electrochemical Immunosensor for Rapid and Sensitive Determination of Estradiol. *Anal. Chim. Acta* **2012**, *743*, 117–124. https://doi.org/10.1016/j.aca.2012.07.002.

(3) Dai, Z.; Yan, F.; Yu, H.; Hu, X.; Ju, H. Novel Amperometric Immunosensor for Rapid Separation-Free Immunoassay of Carcinoembryonic Antigen. *J. Immunol. Methods* **2004**, *287* (1–2), 13–20. https://doi.org/10.1016/j.jim.2004.01.012.

(4) Jolly, P.; Formisano, N.; Tkáč, J.; Kasák, P.; Frost, C. G.; Estrela, P. Label-Free Impedimetric Aptasensor with Antifouling Surface Chemistry: A Prostate Specific Antigen Case Study. *Sensors Actuators, B Chem.* **2015**, *209*, 306–312. https://doi.org/10.1016/j.snb.2014.11.083.

(5) Cecchetto, J.; Carvalho, F. C.; Santos, A.; Fernandes, F. C. B.; Bueno, P. R. An Impedimetric Biosensor to Test Neat Serum for Dengue Diagnosis. *Sensors Actuators, B Chem.* **2015**, *213*, 150–154. https://doi.org/10.1016/j.snb.2015.02.068.

(6) Cavalcanti, I. T.; Guedes, M. I. F.; Sotomayor, M. D. P. T.; Yamanaka, H.; Dutra, R. F. A Label-Free Immunosensor Based on Recordable Compact Disk Chip for Early Diagnostic of the Dengue Virus Infection. *Biochem. Eng. J.* **2012**, *67*, 225–230. https://doi.org/10.1016/j.bej.2012.06.016.

(7) Dias, A. C. M. S.; Gomes-Filho, S. L. R.; Silva, M. M. S.; Dutra, R. F. A Sensor Tip Based on Carbon Nanotube-Ink Printed Electrode for the Dengue Virus NS1 Protein. *Biosens. Bioelectron.* **2013**, *44* (1), 216–221. https://doi.org/10.1016/j.bios.2012.12.033.

(8) Santos, A.; Bueno, P. R.; Davis, J. J. A Dual Marker Label Free Electrochemical Assay for Flavivirus Dengue Diagnosis. *Biosens. Bioelectron.* **2018**, *100*, 519–525. https://doi.org/10.1016/j.bios.2017.09.014.

(9) Sinawang, P. D.; Rai, V.; Ionescu, R. E.; Marks, R. S. Electrochemical Lateral Flow Immunosensor for Detection and Quantification of Dengue NS1 Protein. *Biosens. Bioelectron.* **2016**, *77*, 400–408. https://doi.org/10.1016/j.bios.2015.09.048.

(10) Parkash, O.; Yean, C. Y.; Shueb, R. H. Screen Printed Carbon Electrode Based Electrochemical Immunosensor for the Detection of Dengue NS1 Antigen. *Diagnostics* **2014**, *4* (4), 165–180. https://doi.org/10.3390/diagnostics4040165.

(11) Nawaz, M. H.; Hayat, A.; Catanante, G.; Latif, U.; Marty, J. L. Development of a Portable and Disposable NS1 Based Electrochemical Immunosensor for Early Diagnosis of Dengue Virus. *Anal. Chim. Acta* **2018**, *1026*, 1–7. https://doi.org/10.1016/j.aca.2018.04.032.

(12) Lim, J. M.; Kim, J. H.; Ryu, M. Y.; Cho, C. H.; Park, T. J.; Park, J. P. An Electrochemical Peptide Sensor for Detection of Dengue Fever Biomarker NS1. *Anal. Chim. Acta* **2018**, *1026*, 109–116. https://doi.org/10.1016/j.aca.2018.04.005.

(13) Dutra, R. F.; M Silva, A. C.; Saade, J.; Izabel Guedes, M. F.; Cordeiro, M. T. *A Carbon Ink Screen-Printed Immunoelectrode for Dengue Virus NS1protein Detection Based On Amine Gold Nanoparticles*; 2018.

(14) Silva, M. M. S.; Dias, A. C. M. S.; Silva, B. V. M.; Gomes-Filho, S. L. R.; Kubota, L. T.; Goulart, M. O. F.; Dutra, R. F. Electrochemical Detection of Dengue Virus NS1 Protein with a Poly(Allylamine)/Carbon Nanotube Layered Immunoelectrode. *J. Chem. Technol. Biotechnol.* **2015**, *90* (1), 194–200. https://doi.org/10.1002/jctb.4305.

(15) Alcon, S.; Talarmin, A.; Debruyne, M.; Falconar, A.; Deubel, V.; Flamand, M. Enzyme-Linked Immunosorbent Assay Specific to Dengue Virus Type 1 Nonstructural Protein NS1 Reveals Circulation of the Antigen in the Blood during the Acute Phase of Disease in Patients Experiencing Primary or Secondary Infections. *J. Clin. Microbiol.* **2002**, *40* (2), 376–381. https://doi.org/10.1128/JCM.40.2.376-381.2002.

(16) Muller, D. A.; Depelsenaire, A. C. I.; Young, P. R. Clinical and Laboratory Diagnosis of Dengue Virus Infection. *J. Infect. Dis.* **2017**, *215* (S2), S89–S95. https://doi.org/10.1093/infdis/jiw649.

1. * Corresponding author.

   E-mail addresses: [zhen.zhang@angstrom.uu.se](mailto:zhen.zhang@angstrom.uu.se) (Zhen Zhang) [↑](#footnote-ref-1)
